# Characterization of the initial complaint and care pathways prior to diagnosis in very young sporadic Alzheimer’s disease

**DOI:** 10.1186/s13195-021-00829-0

**Published:** 2021-04-29

**Authors:** Pauline Olivieri, Lorraine Hamelin, Julien Lagarde, Valérie Hahn, Elodie Guichart-Gomez, Carole Roué-Jagot, Marie Sarazin

**Affiliations:** 1 Department of Neurology of Memory and Language, GHU Paris Psychiatry and Neurosciences, Hôpital Sainte Anne, 1 rue Cabanis, F-75014 Paris, France; 2 Université de Paris, F-75006 Paris, France; 3 Université Paris-Saclay, BioMaps, CEA, CNRS, Inserm, F-91401 Orsay, France

**Keywords:** Young-Alzheimer’s disease, Initial complaint, Diagnosis

## Abstract

**Background:**

Very-early-onset Alzheimer’s disease (young-AD) differentiates from late-onset AD (old-AD) by a predominant involvement of the parietal neocortex leading to atypical presentations. The diagnosis of AD is often not the first to be mentioned in such young patients.

**Methods:**

We retrospectively reviewed the initial complaint and care pathways of 66 sporadic young-AD (age < 62) and 30 old-AD patients (age > 65) and compared their neuropsychological profiles at the time of diagnosis (based on clinical-biological criteria) with 44 amyloid-negative controls.

**Results:**

The initial complaint of young-AD was non-cognitive and mimicked a burnout in 32% of cases. Their main cognitive complaints were memory (38% vs 87% in old-AD) and language (17% vs 13%) impairment. The referral to a psychiatrist prior to AD diagnosis was more frequent in young-AD than in old-AD (26% vs 0%). At the time of diagnosis, young-AD were at a more severe stage of dementia than old-AD (24% vs 10% with CDR ≥ 1) but had less anosognosia.

**Conclusions:**

Better identifying the initial signs of very-early-onset AD is crucial to improve the early diagnosis and develop new treatments.

## Background

Two main clinical features differentiate early-onset Alzheimer’s disease (young-AD) from late-onset AD (old-AD): the frequency of atypical phenotypes and the rapidity of clinical decline. Aside from the common typical amnestic presentation, young-AD patients have more often than older AD patients an atypical non-amnestic syndrome with executive, language, or visuo-spatial dysfunction [1, 2]. These phenotypic variants are explained by the location of the cortical damage: in young-AD, the lesions predominantly affect the temporo-parietal cortices with a relative sparing of the hippocampi, whereas in old-AD, a greater medial temporal lobe atrophy is observed, leading to severe amnesia [3–5]. In patients with an atypical non-amnestic presentation, the diagnosis of AD is possible by using pathophysiological biomarkers such as cerebrospinal fluid (CSF) biomarkers or amyloid/tau positron emission tomography (PET) imaging.

Age also plays a role in the rapidity of the clinical progression, the rate of cognitive decline being higher in young than in older AD patients, suggesting a more aggressive disease [1].

The atypical phenotypes in young subjects lead to a delayed diagnosis of young-AD [1]. Combined with the rapid progression of cognitive dysfunction make it more difficult to include these patients in therapeutic trials, as their symptoms are often too pronounced at the time of diagnosis.

Little is known about the initial complaint of young-AD, particularly for patients who still have a professional activity. This information is however of utmost importance to better detect the earliest signs of the disease.

In the present study, we aimed to retrospectively characterize the initial complaint (at the time of the first symptoms) and the care pathways of young-AD patients with or without professional activity, and to compare their neuropsychological profiles at diagnosis with those of old-AD patients. We hypothesized that beyond the purely cognitive complaint affecting memory or language, which is usually reported in old-AD, atypical initial complaints could be identified in young-AD patients, especially in the workplace.

## Methods

### Study design and population

**Fig. 1:**
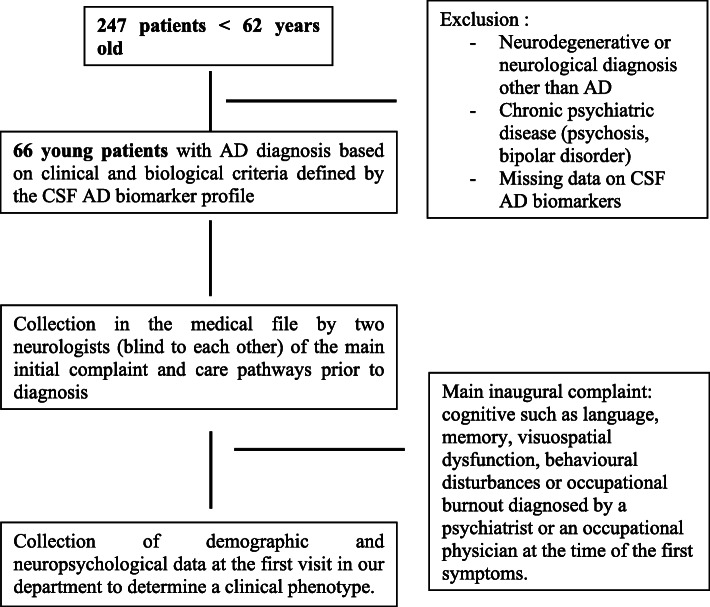
Identification of the initial symptoms and care pathways in very-young AD patients

We retrospectively reviewed the files of all patients younger than 62 referred to the Department of Neurology of Memory and Language at Sainte Anne Hospital in Paris from January 2017 to March 2020 (*n* = 247) (Fig. 1). Among them, 66 patients had a diagnosis of AD based on clinical and biological criteria defined by the CSF AD biomarker profile. We have chosen the age of 62 years, in order to target patients likely to be in active employment, as 62 is the legal age for retirement in France. In addition, 30 old-AD patients with a clinical-biological diagnosis (CSF AD biomarker profile) and a group of 44 controls (16 younger than 62 and 28 older controls) with a negative PiB-PET imaging were included. In addition, 15 AD patients (2 young-AD and 13 old-AD) had a PiB-PET imaging, which was positive in all cases.

Two neurologists, blind to each other, collected retrospectively in the medical file the main initial complaints of all patients, which were classified as (1) cognitive including language, memory, visuospatial dysfunction, or behavioral disturbances or (2) occupational burnout diagnosis according to the World Health Organization ICD11 definition [6]. They also collected their care pathways before they were referred to our department. When there was more than one complaint, the instruction was to consider as the main complaint the one leading to the neurological consultation and being at the forefront of the interview with the patient and his/her caregiver. For all types of complaints other than behavioral, the patient’s and the caregiver’s statements were concordant. For behavioral complaints, which could be more subjective, we only considered the impression of the caregiver. The diagnosis of occupational burnout syndrome was made by a psychiatrist or an occupational physician, at the time of the first symptoms, before the patient was referred to our department [6]. It was characterized by a feeling of reduced professional efficacy and energy depletion or exhaustion, leading to a severe anxiety, in the absence of cognitive neurological symptoms [6]. The diagnosis of burnout was retained when no other neurological cognitive disorder was reported by the patient, family, or the psychiatrist or occupational physician. All patients performed the same neuropsychological battery at the time of diagnosis. In addition, we assessed social life changes and cognitive (memory) anosognosia by the Cambridge Behavioural Inventory Revised Scale (CBI-R) [7] and the Mc Nair scale, which were filled both by the patients and their caregiver.

All controls provided written informed consent as part of ongoing research protocols (Imabio3 and Shatau7-Imatau studies). In accordance with the French legislation, patients for whom clinical and CSF data were generated during routine clinical workup and their relatives were informed that individual data could be used in clinical research studies and they signed a specific consent form (MA-D20-R56 study).

### Statistical analysis

All statistical analyses were performed with SPSS® 26.0 (SPSS Inc., Chicago, IL, USA). A chi-square or Fisher’s exact test was performed for group comparisons of categorical data. A rank sum test or *t*-test was used for analyses of continuous variables. The results of quantitative variables are presented as means ± standard deviations (SD). For dichotomous variables, numbers and calculated percentages are presented. *P*-values < 0.05 were considered statistically significant.

## Results

### Initial complaint and care pathways before diagnosis (Table 1)

The initial complaint of young-AD patients was memory (38%), language (17%), visuo-spatial (6%), or behavioral (7%) impairment. In 32% of young-AD patients, the initial complaint was an occupational burnout-like syndrome. The diagnosis of burnout was made by a psychiatrist or an occupational physician for 80% of these cases, in the absence of overt language, memory, gestural, visuo-spatial, neurological behavioral disorders or even other neurological signs. For these patients, families did not report any specific cognitive symptom. In the sub-group of young-AD patients having a professional activity (*n* = 46), burnout was the initial complaint in 46% of cases. In the old-AD patients, the initial complaints were mainly memory (87%) and language (13%) impairment. Fifty-two percent of young-AD patients with a burnout syndrome were initially referred to a psychiatrist (vs 13% of the young-AD patients with an initial cognitive complaint) and 28% to an occupational physician.

**Table 1:**
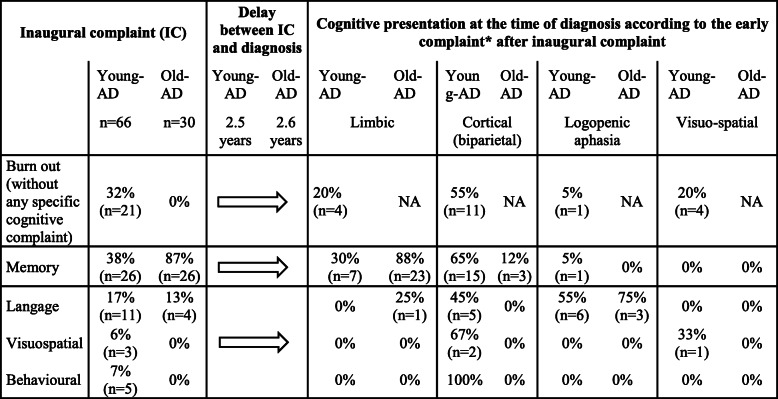
Inaugural complaint and cognitive phenotype at diagnosis in young and old-AD patients

*Data available for 61 patients with young-AD

Four cognitive presentations have been identified from the results of the neuropsychological assessments:

-Limbic characterized by hippocampal amnestic syndrome, [8]

-Biparietal dysfunction characterized by a visuospatial deficit, dyspraxia, dysgraphia, logopenic aphasia, and deficit of auditory-verbal short-term memory [2, 9]

-Logopenic variant primary progressive aphasia according to the clinical criteria of Gorno-Tempini et al. 2011 [10].

-Visual spatial dysfunction, known as posterior cortical atrophy (PCA) or “Benson’s disease” characterized by oculomotor apraxia, optic ataxia, dressing apraxia, environmental disorientation, abnormal anti-saccades, neglect, constructional difficulty, simultanagnosia, visual agnosia, and prosopagnosia [11, 12].

### Cognitive phenotype and neuropsychological evaluation at the time of diagnosis

The diagnosis was made more than 2 years after the first reported complaint. A phenotype of cognitive biparietal dysfunction (visuospatial deficit, dyspraxia, dysgraphia, logopenic aphasia and deficit of auditory-verbal short term memory [2, 9]) was the most common, observed in 55% and 64% of young-AD patients with and without burnout (see Table 1). The comparisons of the neuropsychological scores between young-AD, old-AD, and, respectively, young and old controls are detailed in Table 2. Young-AD patients presented with a more severe cognitive impairment, a greater loss of autonomy assessed by the Clinical Dementia Rating (CDR) scale (40% of young-AD patients had a CDR ≥ 1 versus 10% in old-AD patients), and less anosognosia compared to old-AD. No clinical or neuropsychological difference was observed between young-AD with and without an initial burnout, except for educational level, which tended to be higher in the former (Table 2).

**Table 2:** Neuropsychological assessment in young-AD presenting with and without an initial burnout like syndrome (BO), old-AD, young and old controls (YC, OC)

				Old-AD (*n* = 30)	YC (*n* = 16)	OC (*n* = 28)	*P* (cdr)°
		Young-AD (*n* = 66)					
		BO (*n* = 21)	No BO (*n* = 45)				
Age (years)		55.1 (6.6)	57.8 (3.9)	74.2 (4.8)	53.8 (10)	71 (4.1)	**< 0.001**
Age of onset		52.62 (6.4)	54.5 (4.1)	71.5 (4.8)	NA	NA	**< 0.001**
Educational level^#^	2–3	4.7% (*n* = 1)	24.4% (*n* = 11)	NA	NA	NA	
	3–4	14.3% (*n* = 3)	26.6% (*n* = 12)	NA	NA	NA	
	5–6	81% (*n* = 17)	50% (*n* = 22)	NA	NA	NA	
History of depression		4.8% (*n* = 1)	6.7% (*n* = 3)	NA	NA	NA	
***Neuropsychological assessment***							
CDR	0.5	76.2% (*n* = 16)	51.1% (*n* = 23)	90%	NA	NA	**0.007**
	≥ 1	23.8% (*n* = 5)	48.9% (*n* = 22)	10%	NA	NA	
Global cognitive efficiency	MMSE	20.1 (4.1)	17.3 (5.8)	24 (3.7)	29.2 (1.1)**	29.2 (0.8)**	**< 0.001**
	Spatiotemporal orientation	7.3 (1.9)	6.3 (2.8)	8.0 (2.5)	9.9 (0.3)**	9.9 (0.3)**	0.12
Episodic Memory	FCSRT Immediate recall (16)	8.2 (5.2)	8.4 (4.4)	11.9 (3.5)	15.7 (0.5)**	15.8 (0.5)**	**0.002**
	FCSRT Free recall (48)	13.9 (12.4)	12.1 (9.7)	12.6 (7)	34.3 (4.9)**	32.6 (4)**	0.07
	FCSRT Total recall (48)	27.7 (15.3)	26.4 (13.5)	29.4 (13)	47.5 (0.7)**	47.3 (1)**	0.5
	ROCF recall (36)	6.2 (3)	8.2 (7.1)	8.1 (8)	19.4 (5.1)**	19.4 (6)**	0.8
Attention and working memory	Verbal backward digit span	5 (1.2)	4.4 (1.3)	5.3 (0.9)	6.5 (0.9)**	5.9 (1.3)	**0.023**
	Verbal forward digit span	2.8 (0.8)	2.4 (1.1)	4 (1.1)	4.7 (1.1)**	4.8 (1.2)*	**< 0.001**
	Visual backward digit span	3.4 (1.8)	3.1 (1.5)	4.5 (1.3)	NA	NA	0.15
	Visual forward digit span	2.7 (1.2)	2.3 (1.7)	3.5 (1.6)	NA	NA	**0.001**
Executive functions	Literal Verbal fluency (2 min)	14.5 (9.3)	9.1 (13.9)	16.5 (8.2)	36.7 (7.6)**	35.2 (10)**	**0.02**
	Categorial Verbal fluency (2 min)	18.2 (9.6)	13.9 (6.3)	21.9 (7.6)	25.5 (8.3)**	24.3 (7.3)**	**0.003**
	TMTB-A	111.6 (54.9)	154.3 (72.8)	103.3 (76)	36.3 (23.5)**	40.6 (24.9)**	0.15
Instrumental functions	Kinesthetic praxies	21.4 (7.7)	21.6 (8.1)	26.1 (5.2)	NA	NA	0.059
	Ideomotor praxis (without signification)	20 (10.2)	20.6 (10.6)	27.1 (5.6)	29.6 (0.5)**	28.9 (1.4)**	**0.017**
	Ideomotor praxis (action mimic)	23.4 (7.5)	24.8 (6.5)	27.5 (5)	NA	NA	0.18
	Naming (80)	34.4 (5.6)	30.5 (9.3)	37.7 (7.4)	40 (0)**	28.9 (1.4)	**0.007**
	Copy of the Rey figure (36)	20.5 (15.4)	30.1 (12)	34.4 (1.3)	34.6 (2.1)**	59.2 (1.7)	**0.008**
Anosognosia	Functional/social ^&^	7.7 (27)		25 (26)	NA	NA	**0.01**
	Memory ^&&^	9.6 (21)		20 (24)	NA	NA	0.1

Data are mean (SD). *p < 0.05 and **p < 0.001, in comparison with controls

*p* (cdr)° comparison between young-AD and old-AD adjusted with CDR score

With Prof. act.: with professional activity

*CDR* Clinical Dementia Rating Scale, *FCSRT* Free and Cued Selective Reminding test, *ROCF* Rey-Osterrieth Complex Figure, *TMT* Trail Making test (A and B)

^#^Educational level was quoted as follows: 1, no diploma; 2–3, 5 years of scholarship; 4–5, from 9 to 12 years of education; 6–7, more than 12 years of education

^&^Difference between the score of the Cambridge Battery Inventory (CBI) assessed by the caregiver and by the patient. ^&&^Difference between the score of the Mac Nair scale assessed by the caregiver and by the patient

## Discussion

Young-AD is the most common early-onset neurodegenerative disease and presents less commonly with memory deficits and more frequently with focal cortical dysfunction, which makes the diagnosis challenging. In our cohort, 68% of the young-AD patients (younger than 62 years) had a purely cognitive initial complaint and were referred primarily to a neurologist. Interestingly, in a third of our young-AD patients, the initial complaint was atypical and led to the initial diagnosis of a burnout syndrome. Among the young-AD patients with a professional activity (70%), a burnout-like syndrome was the first diagnosis in almost half of the cases. These patients had an inability to carry out concurrent professional tasks, leading to a reduction of professional efficacy and a severe anxiety, in the absence of overt language, memory, gestural, visuo-spatial disorders, or other neurological signs. They were conscious of their difficulties and tried to compensate, which led to work overload, mental exhaustion, and personal depreciation. Their relatives did not report any specific cognitive abnormality. Most of these patients were treated by a psychiatrist during several months, before being referred to a neurologist. It is crucial to detect this type of situation as early as possible, in order to offer the most appropriate care, such as specific medication, rehabilitation, and adaptation of the workspace when possible, and also to avoid the prescription of contraindicated treatment such as anticholinergic antidepressants.

As expected, in old-AD patients, the initial complaint was about memory (87%), or language, with a lack of words (13%).

The time between the first symptoms and the first neuropsychological assessment was more than 2 years, without any significant difference between old-AD and young-AD. A greater delay of diagnosis in young-AD than old-AD has however been reported previously, [13] but could not be attributed to anosognosia, which is less pronounced in young-AD patients.

Young-AD presented with a more severe cognitive impairment at diagnosis compared to old-AD, especially with regard to instrumental functions (language, gestural praxis, visuo-spatial abilities), and working memory, resulting in a greater loss of autonomy and lower MMSE scores.

Compared to old-AD, neuroimaging studies showed that young-AD patients may have a relative preservation of hippocampal volume and a predominant parietal atrophy, [3, 4] with a more severe parietal hypometabolism, [14] which is congruent with a greater percentage of atypical presentations in these young patients. The extent and distribution of tau pathology measured by PET also differed between young-AD and old-AD, with tau aggregation in widespread neocortical regions (prefrontal and parietal cortex) in young-AD while the pattern of tau deposition was more confined to the temporal regions in old-AD [5].

Burnout-like syndrome could be due to an early alteration of the fronto-parietal connectivity. MRI studies suggest that functional connectivity changes differ in young-AD and old-AD, young-AD being mainly driven by an early involvement of fronto-parietal networks [15]. Fronto-parietal circuit alterations contribute to impairments in central executive network, top-down attentional control, and working memory [16]. Progressive changes of neural networks are present before neuronal loss and regional atrophy [17] and could contribute to the occurrence of burnout-like syndromes before the onset of more classic cortical cognitive signs. The hypotheses regarding the anatomical underpinnings of the burnout-like syndrome in these patients will need to be tested in dedicated studies including imaging data.

### Limitations

The present study has some limitations, particularly its retrospective nature. This is however inherent to the data studied, which can only be collected retrospectively. In order to limit selection bias, the patient’s initial complaint was collected by two neurologists blind to each other, whose interpretations were all congruent.

## Conclusions

Early symptoms like occupational burnout-like syndrome could be under-recognized in young-AD and could possibly be underlain by a working memory deficit. It is crucial to consider and further study these early symptoms to avoid delayed diagnosis, which often impacts the quality of patients’ care and compromises their chances of participating in therapeutic trials, due to already advanced cognitive and functional alteration at the time of diagnosis.

## Data Availability

The dataset used during the current study is available from the corresponding author on reasonable request.

## Abbreviations

AD: Alzheimer’s disease
CSF: Cerebrospinal fluid
CDR: Clinical Dementia Rating
